# Two cases of dupilumab-associated conjunctivitis with high expression of IL-8 mRNA on the ocular surface: a case report

**DOI:** 10.1186/s13223-022-00727-6

**Published:** 2022-10-02

**Authors:** Rumi Adachi, Jun Shoji, Akira Hirota, Akiko Tomioka, Yukiko Tonozuka, Noriko Inada, Satoru Yamagami

**Affiliations:** Division of Ophthalmology, Department of Visual Sciences, Nihon University School of Medicine, 30-1 Oyaguchi-Kamichou, Itabashi-ku, Tokyo, 173-8610 Japan

**Keywords:** Dupilumab, Conjunctivitis, Eotaxin-2, Interleukin-8

## Abstract

**Background:**

Dupilumab-induced ocular surface disease (DIOSD) has been reported in patients with atopic dermatitis treated with dupilumab, and has been recognized as an adverse event of dupilumab. Our objective was to describe two cases of DIOSD with alterations in eotaxin-2 and interleukin (IL)-8 messenger ribonucleic acid (mRNA) expression on the ocular surface.

**Case presentation:**

In the ocular surface test, specimens were collected from the patient's ocular surface, and eotaxin-2 and IL-8 mRNA levels in the specimens were measured using real-time polymerase chain reaction. The clinical score of ocular surface findings was quantified using a 5-5-5 exacerbation grading scale for allergic conjunctivitis. The first case was of a 27-year-old man who developed DIOSD 3 months after starting treatment with dupilumab injection for atopic dermatitis. After 5 weeks of topical instillation of tacrolimus ophthalmic suspension, the clinical score of ocular surface findings improved and IL-8 and eotaxin-2 mRNA expression levels gradually decreased. The second patient was a 55-year-old man who developed DIOSD 11 weeks after the start of treatment with dupilumab injection for atopic dermatitis. Four weeks after starting ophthalmological treatment with tacrolimus ophthalmic suspension, his clinical scores on ocular surface findings improved and IL-8 mRNA expression levels decreased. The ocular surface test in this case revealed increased expression levels of IL-8 mRNA on the ocular surface at the onset of DIOSD, which decreased with the improvement of objective findings.

**Conclusions:**

DIOSD, which has been successfully treated with tacrolimus ophthalmic suspension, may involve IL-8-related inflammation in addition to type 2 inflammation.

## Background

Atopic dermatitis (AD) is a chronic inflammatory disease characterized by severe skin irritation and pruritic, erythematous, and scarring skin lesions. The major immunological pathogenesis of AD is understood to be the T helper type 2 (Th2) response, including the cytokine effects of interleukin (IL)-4 and IL-13, with additional roles for Th17, Th22, and Th1 cytokines in certain disease subtypes. The cytokines produced by ILC2, including IL-13, are also involved in the immunological pathogenesis of AD. Ocular complications such as atopic keratoconjunctivitis (AKC), keratoconus, cataracts, and retinal detachment are known to develop in severe AD.

Dupilumab, an anti-human alpha subunit of IL-4 and IL-13 receptor monoclonal antibody, is the only dual inhibitor of IL-4 and IL-13 signaling. In addition, dupilumab therapy has been approved for indications including atopic dermatitis, bronchial asthma, and rhinosinusitis with nasal polyposis in Japan, and reduced Th2 responses in these allergic diseases [1]. Conjunctivitis, blepharitis, and keratitis have been reported in patients with AD treated with dupilumab, and have been recognized as adverse events of dupilumab [2]. A previous clinical trial on dupilumab in patients with AD reported an 8.5% (78 of 920 patients) incidence of conjunctivitis as an adverse event [3]. Dupilumab-induced blepharitis and conjunctivitis have a wide variety of clinical phenotypes including moderate to severe conjunctivitis [4], follicular conjunctivitis [5], giant papillary conjunctivitis [6, 7], blepharoconjunctivitis [8], cicatrizing conjunctivitis [9], and corneal limbitis [5]. Therefore, ocular surface diseases that occur during dupilumab treatment have been names dupilumab-induced ocular surface diseases (DIOSDs) [10, 11]. However, the detailed pathogenesis of DIOSD is not yet fully understood.

In this case report, we conducted an observational study using clinical scores [12] and ocular surface test [13, 14] to analyze the pathogenesis of inflammation occurring in DIOSD. We examined messenger ribonucleic acid (mRNA) expression levels of IL-8 as a neutrophil-related marker [15] and eotaxin-2/CCL24 as an eosinophil-related marker [16] for DIOSD in patients with atopic dermatitis undergoing treatment with dupilumab.

## Case presentation

### Clinical severity score

To evaluate the ophthalmological clinical severity score, we used our recently reported 5-5-5 exacerbation grading scale for allergic conjunctival diseases [12]. In addition, the Eczema Area Severity Index (EASI) [17] and Patient-Oriented Eczema Measure (POEM) scores [18] were used to evaluate the severity and activity of AD.

### Ocular surface test

The ocular surface test is an ophthalmological clinical test that combines specimen collection by impression cytology and the measurement of cytokine and chemokine mRNA levels expressed on the ocular surface by quantitative reverse transcription polymerase chain reaction (qRT-PCR) [13]. This study was approved by the Institutional Review Board of Nihon University School of Medicine (approval number: RK-190709-2), and written informed consent was obtained from all patients prior to testing.

#### Impression cytology

Using the impression cytology method, specimens for qRT-PCR were collected from the upper palpebral conjunctiva. Specimens were collected by pressing a filter paper disk made by excising the tip of a Schirmer test paper (Tear Production Measuring Strips; AYUMI Pharmaceutical Corporation, Tokyo, Japan) against the unanesthetized palpebral conjunctiva. Messenger RNA (mRNA) was extracted from the filter paper disc using a MagLEAD^®^ automated nucleic acid extraction system (Precision System Science, Chiba, Japan).

#### Real-time reverse transcription polymerase chain reaction

Real-time RT-PCR was performed using the GeneSoc^®^ microfluidic real-time PCR system (KYORIN Pharmaceutical, Tokyo, Japan), TaqMan gene expression assay (Life Technologies), and predesigned primers/probes, including Hs99999034_m1 (IL-8) and Hs00171082_m1 (eotaxin-2) (Life Technologies Japan, Tokyo, Japan). The target cycle threshold (Ct) values were normalized to those of GAPDH (Hs99999905_m1) from the same sample. The relative expression levels of each target gene were determined using the ∆∆CT method. The reference value for each mRNA expression level was set as 1.

### Case series

In the two cases, the patients diagnosed with DIOSD had complained of subjective exacerbation of hyperemia and had findings of conjunctivitis on slit-lamp microscopy during the period of dupilumab treatment.

#### Case 1

**Fig. 1:**
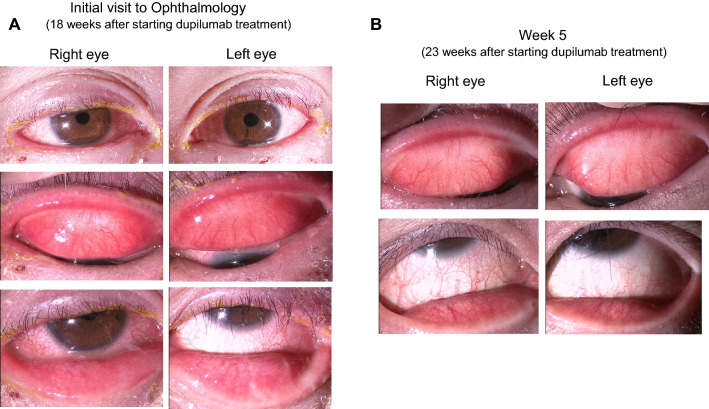
Photographs of blepharitis and conjunctivitis in case 1. **a** At the initial ophthalmology visit, posterior blephalitis including meibomian gland inflammation, conjunctivitis with velvety appearance of palpebral conjunctivitis, severe bulbal hyperemia, and mucopurulent discharge were observed. **b** Five weeks after starting ophthalmologic treatment, clinical findings of blepharoconjunctivitis were improved

**Fig. 2:**
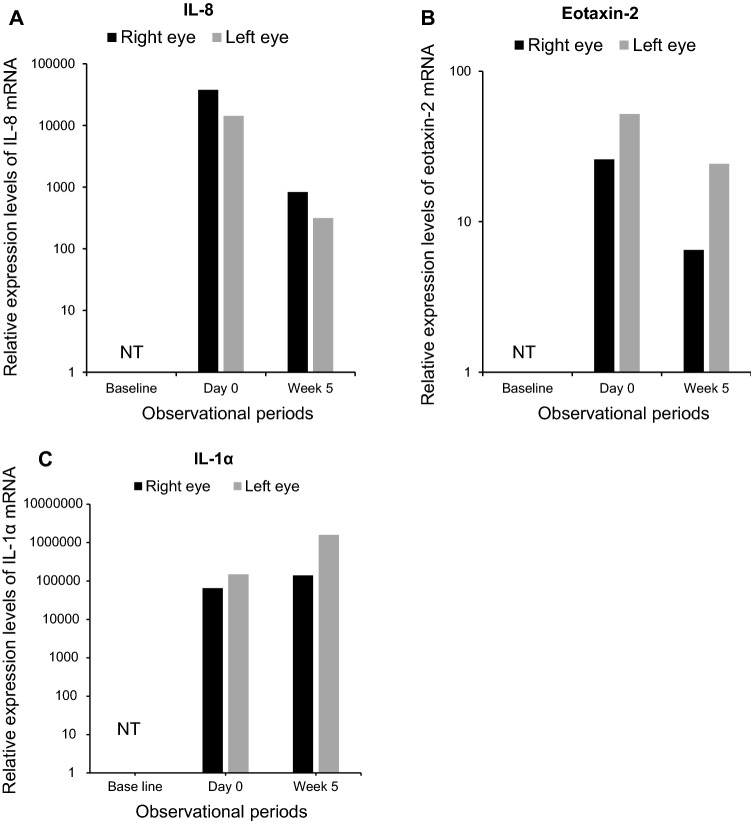
Results of ocular surface test for IL-8, eotaxin-2, and IL-1α in case 1. **a** Relative expression of IL-8 mRNA on the ocular surface. IL-8 mRNA levels peaked in both eyes at the onset of conjunctivitis (day 0) and decreased as conjunctivitis became milder. **b** Relative expression levels of eotaxin-2 mRNA on the ocular surface. Eotaxin-2 mRNA levels were mildly elevated at the onset of conjunctivitis. NT, not tested. **c** Relative expression levels of IL-1α mRNA on the ocular surface. The expression level of IL-1α mRNA did not show a strong correlation with the clinical findings of conjunctivitis

A 27-year-old man had moderate-severe AD (EASI score was 27.7 points at the beginning of dupilumab treatment), presented to ophthalmology department of our hospital complaining progressively increasing hyperemia, blepharitis, and epiphora in his bilateral eyes 3 months after starting dupilumab treatment for AD (day 0). Laboratory examinations before initiation of dupilumab treatment revealed peripheral blood eosinophil percent of 15.0%, and serum thymus and activation-regulation chemokine (TARC) level of 623 pg/mL. The patient had been on treatment with 300 mg of subcutaneously injected dupilumab (Dupixent, Sanofi K.K., Tokyo, Japan) every 2 weeks. At the initial visit to our department, her EASI score was 3.2 points. Slit-lamp examination revealed atopic blepharitis, velvety papillary proliferation of the upper palpebral conjunctiva, and severe hyperemia of the bulbar conjunctiva. The ophthalmological clinical severity score at the initial visit was 134. He was diagnosed with DIOSD and treated with 0.1% tacrolimus hydrate ointment once per day for atopic blepharitis, tacrolimus ophthalmic suspension twice per day, and 0.5% cefmenoxime ophthalmic solution twice per day for conjunctivitis. Five weeks after starting ophthalmological treatment (week 5), the objective findings of blepharitis and conjunctivitis improved (Fig. 1), and the ophthalmological clinical severity score decreased to 13 points. The results of the ophthalmological clinical severity scores and ocular surface tests are shown in Table 1 and Fig. 2. An ocular surface test at the initial ophthalmology visit showed markedly elevated IL-8 mRNA levels in both eyes. At week 5, the IL-8 mRNA levels had decreased more than 50-fold. The eotaxin-2 mRNA levels showed a similar trend in the ocular surface tests, but the changes during the treatment period were less pronounced. The IL-1α mRNA expression levels were largely unrelated to the severity of conjunctivitis.

**Table 1:** Clinical scores of 5-5-5 exacerbation grading scale, EASI, and POEM

Case	Visit	5-5-5 exacerbation grading scale (points)		EASI score (points)	POEM score (points)
		Right eye	Left eye		
Patient 1	Start of dupilumab injection	NT	NT	27.7	23
	Week 18 (Day 0^a^)	134	134	3.2	12
	Week 23	13	13	4	9
Patient 2	Start of dupilumab injection	14	14	41.2	28
	Week 11 (Day 0^a^)	124	124	5.65	1
	Week 15	24	24	4.1	0

*NT* not tested

^a^Day 0 means the first visit to ophthalmologist after the onset of conjunctivitis

#### Case 2

**Fig. 3:**
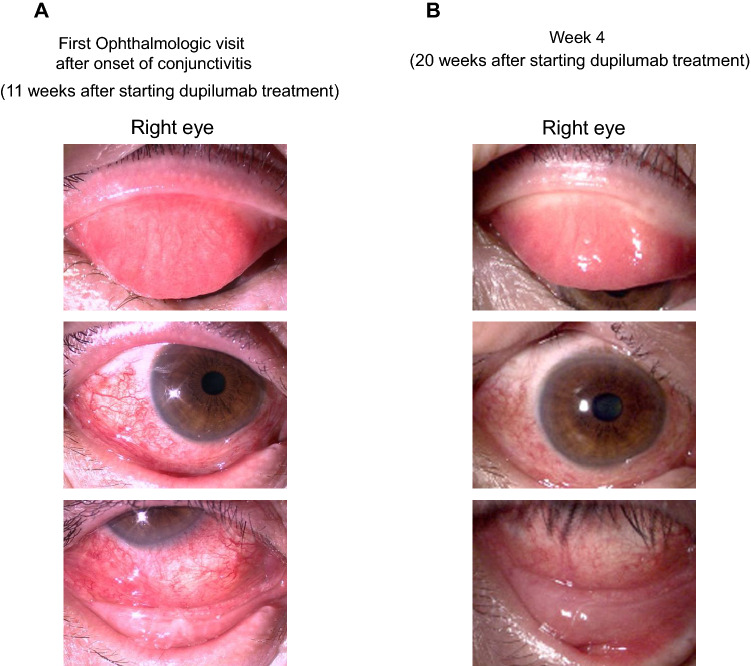
Photographs of blepharitis and conjunctivitis in case 2. **a** At the first ophthalmology visit after the onset of conjunctivitis, conjunctivitis with a velvety appearance of palpebral conjunctivitis and severe bulbal hyperemia were observed. **b** Nine weeks after starting ophthalmologic treatment, the clinical findings of conjunctivitis mostly resolved, but mild hyperemia continued

**Fig. 4:**
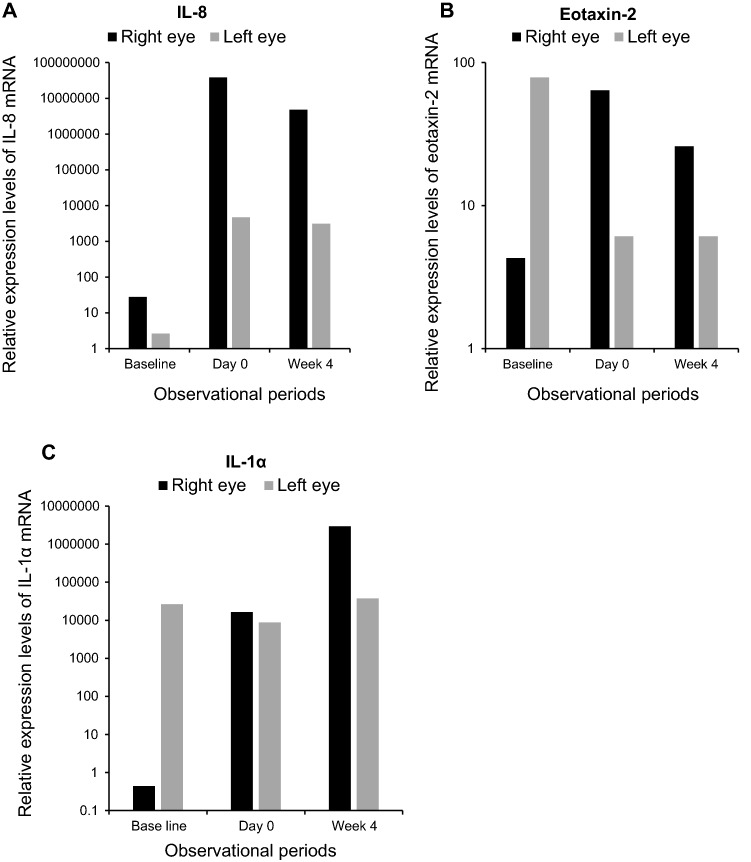
Results of ocular surface test for IL-8, eotaxin-2, and IL-1α in case 2. **a** Relative expression of IL-8 mRNA on the ocular surface. The IL-8 mRNA levels peaked in both eyes at the onset of conjunctivitis (day 0) and decreased thereafter. **b** Relative expression levels of eotaxin-2 mRNA on the ocular surface. Eotaxin-2 mRNA levels were mildly elevated in (right eye) or virtually unchanged (left eye) with the onset of conjunctivitis. **c** Relative expression levels of IL-1α mRNA on the ocular surface. The expression level of IL-1α mRNA did not show a strong correlation with the clinical findings of conjunctivitis

A 55-year-old man with severe AD (EASI score of 41.2 points) developed AKC and continued AKC treatment in the ophthalmology department of our hospital. He was started on dupilumab treatment by a dermatologist owing to the severity and refractoriness of his AD. Laboratory examinations before initiation of dupilumab treatment revealed peripheral blood eosinophil percent of 5.5% and a serum TARC level of 216 pg/mL. Before dupilumab administration, his clinical scores were recorded, and specimens were collected from both eyes for the ocular surface test. The ophthalmological clinical severity score was determined to be 113 points. He had been on treatment with 300 mg of subcutaneously injected dupilumab (Dupixent, Sanofi K.K., Tokyo, Japan) every 2 weeks. Eleven weeks after the start of dupilumab treatment (day 0), conjunctival hyperemia exacerbated. His objective findings in the lid, conjunctiva, and cornea included atopic blepharitis, velvety papillary proliferation of the upper palpebral conjunctiva predominantly in the right eye, and severe hyperemia of the bulbal conjunctiva (Fig. 3). His ophthalmological clinical severity score in both eyes increased to 124 points. He was diagnosed with DIOSD, and treatment for DIOSD was continued with tacrolimus ointment once a day, which was changed from dexamethasone ointment for atopic blepharitis and tacrolimus ophthalmic solution twice a day for AKC and DIOSD. Four weeks after starting ophthalmological treatment (week 4), the ophthalmological clinical severity score improved to 24 points. The alterations in EASI, POEM, ophthalmological clinical severity score, and ocular surface test results are shown in Table 1 and Fig. 4. At the first ophthalmologic visit after the onset of conjunctivitis, the ocular surface test showed a markedly higher level of IL-8 mRNA compared to baseline in both eyes. At week 4, IL-8 mRNA levels had decreased. The eotaxin-2 mRNA levels were generally low in the ocular surface tests, although mild changes were noted during the course of the treatment. The IL-1α mRNA expression levels were largely unrelated to the severity of conjunctivitis.

## Discussion and conclusions

In our case report on DIOSD, we clarified the clinical characteristics of AD patients with DIOSD using clinical scores and ocular surface tests. Each patient showed blepharitis and severe conjunctivitis was commonly observed, and IL-8 mRNA expression was increased on the ocular surface during the development of DIOSD. Furthermore, ophthalmic treatment with a tacrolimus ophthalmic suspension and tacrolimus ointment is useful for DIOSD.

The clinical findings of DIOSD in our cases were characterized by severe conjunctival hyperemia with conjunctival swelling in the palpebral and bulbar conjunctiva, which was the same as previously reported [4, 5, 10, 11]. However, these conjunctival findings are not specific to DIOSD, and the differential diagnosis of acute exacerbation of AKC is difficult. In the future, specific findings that can be used for a definitive diagnosis of DIOSD should be established.

The results of the ocular surface test in our case revealed increased IL-8 mRNA expression levels in the upper tarsal conjunctiva at the onset of DIOSD. IL-8 is a CXC chemokine involved in neutrophil migration, and the IL-8 mRNA expression level was used in this study as an ocular surface marker of neutrophilic inflammation. Increased IL-8 levels in the tears have been reported in patients with infectious conjunctivitis and trauma [19]. Furthermore, in allergic conjunctival diseases, IL-8 levels have also been reported to be elevated in the tears of patients with giant papillary conjunctivitis and vernal keratoconjunctivitis (VKC) [20]. Aso et al. reported that expression levels of IL-1α, IL-8, IL-16, and eotaxin-2 mRNA were elevated in the ocular surface test of patients with chronic ACD, including AKC and VKC, and that there was a significant correlation between IL-1α and IL-8, and between IL-16 and eotaxin-2. We speculated that two inflammatory systems, eotaxin-2-associated inflammation and IL-8-associated inflammation, are involved in the pathogenesis of chronic allergic conjunctivitis, including AKC and VKC [14]. In addition, Leonaldi et al. demonstrated that ocular surface tests of patients with VKC showed increased mRNA expression of Th2/Th17-signaling families and proinflammatory cytokines [21], suggesting that Th2 and Th17 reactions are key factors in the pathogenesis of chronic allergic diseases. The increase in IL-8 levels in inflammatory tissues is known to be related to innate immunity, Th17 response, histamine stimulation, and reactive oxygen species [22]. It is unclear what triggered the increased IL-8 in our patients, but it is possible that DIOSD causes a different inflammatory response than eosinophilic inflammation induced by Th2 inflammation. Bakker et al. reported that conjunctival biopsies from patients with atopic dermatitis treated with dupilumab showed elevated Th1/Th17 cytokines in the conjunctiva [23].

In contrast, eotaxin-2 mRNA expression, which is used as a marker of eosinophilic inflammation, remained mildly increased during the observation period. These test results indicate that dupilumab injections and tacrolimus eye drop instillation suppress Th2 reactions and eosinophilic inflammation in the conjunctival tissues.

Our case report had several limitations. First, the diagnostic criteria for DIOSD are unclear. In the future, it will be necessary to establish diagnostic criteria for DIOSD in a larger number of patients with AD receiving dupilumab treatment. Second, the clinical tests for ocular surface markers were semi-quantitative. In the future, the results of ocular surface tests should be incorporated into diagnostic criteria as absolute measurements.

In conclusion, DIOSD, which has been successfully treated with tacrolimus ophthalmic suspension, may involve IL-8-related inflammation in addition to type 2 inflammation.

## Data Availability

All data generated or analyzed during this study are included in this published article.

## Abbreviations

ACD: Allergic conjunctival disease
AD: Atopic dermatitis
AKC: Atopic keratoconjunctivitis
DIOSD: Dupilumab-induced ocular surface disease
EASI: Eczema area severity index
IL-8: Interleukin-8
POEM: Patient-oriented eczema measure
RT-PCR: Reverse transcription polymerase chain reaction
VKC: Vernal keratoconjunctivitis
